# Tuberculosis treatment outcomes among pulmonary TB patients attending public hospitals in Kebbi State, Northern Nigeria: a four-year retrospective study

**DOI:** 10.1186/s42269-022-00969-9

**Published:** 2022-12-13

**Authors:** Mohammed Bashar Danlami, Aliyu Basiru, Yahaya Tajjudeen, Abbas Yusuf Bazata, Bashar Haruna Gulumbe, Musa Mohammed

**Affiliations:** 1 Department of Microbiology, Faculty of Sciences, Federal University Birnin Kebbi, P.M.B. 1157, Kalgo, Nigeria; 2 Department of Biological Sciences, Faculty of Sciences, Federal University Birnin Kebbi, P.M.B. 1157, Kalgo, Nigeria; 3 Department of Medical Laboratory Science, Kebbi State College of Health Science and Technology, P.M.B. 9003, Jega, Kebbi State Nigeria

**Keywords:** Successful outcome, Treatment outcome, Pulmonary tuberculosis, Kebbi, Nigeria

## Abstract

**Background:**

In Nigeria, effective case management and evaluation of pulmonary tuberculosis treatment outcomes are an integral part of controlling the spread of infectious diseases. The study reviewed the treatment outcomes of pulmonary tuberculosis and the factors associated with rates of successful and unsuccessful treatment outcomes in the 21 referral hospitals in Kebbi State, Nigeria.

**Methods:**

Documented records of pulmonary tuberculosis patients from January 2018 to December 2021 in 21 Local Area Councils in Kebbi State, Northern Nigeria were reviewed. A structured questionnaire collated the socio-demographic and clinical data from the documented records. Descriptive statistics were used to compute and analyse the outcomes of successful and unsuccessful treatment. Logistic regression models were used to determine the association of socio-demographic and clinical data with the unsuccessful treatment outcomes.

**Results:**

The study reviewed data from 6114 records of TB patients. 1161 (18.9%) started treatment, 963 (82.9%) were males and 198 (17.1%) were females. Of the 1161 patients, 985 (18.2%) had documented treatment outcomes. 932 of 985 (95.1%) had a pulmonary infection. 64 (5.8%) patients with documented treatment outcomes were HIV seropositive. 903 (91.7%) were successfully treated, and 82 (8.3%) failed. Of the patients with failed treatment outcomes, 15 (1.5%) were lost to follow-up, 43 (4.4%) defaulted and 24 (2.4%) died. In the logistic analysis, the odds of unsuccessful treatment outcomes were higher among elderly patients (AOR = 2.00, 95% CI 1.37–2.92), patients with extrapulmonary infections (AOR = 2.40, 95% CI 1.12–5.39), and with old cases of pulmonary TB (AOR = 3.03, 95% CI 1.47–7.19) when compared to their groups.

**Conclusions:**

The study reported a treatment success rate of 91.7% among TB patients attending public hospitals in Kebbi State. The outcome was higher than the projected success rate of 85% set by the WHO. However, one-fourth of the total patients reviewed were not documented for treatment. Therefore, the need to design an appropriate recruitment strategy to identify and enrol those patients for an effective and successful TB control program in Nigeria.

## Background

In 2020, despite the accessibility of highly effective treatment, there were an estimated 10 million new cases of pulmonary tuberculosis worldwide. 5.6 million men, 3.3 million women, 1.1 million children and 1.5–2.0 million deaths worldwide (Chakaya et al. 2021; Harding 2020). The counties with the highest burden of TB cases were in the South-East Asian Region (43%), African countries account for 25% and then the Western Pacific with 18%. Eight out of the 30 endemic countries accounted for two-thirds of the total new cases of TB (Chakaya et al. 2021; Harding 2020). Incidentally, Nigeria falls under this category representing a high burden of new TB cases: a leading cause of mortality rate in the country (Harding 2020; Oladimeji et al. 2021).

To prevent and control the high burden of new TB cases and reduce the mortality rate due to tuberculosis, the WHO recommends a Directly Observed Therapy (DOTS) approach for the case management of persons with active TB disease. The DOTS approach is a standardized short-course chemotherapy strategy effective in the diagnosis, treatment, and control of pulmonary tuberculosis (Harding 2020; Chakaya et al. 2022).

In Nigeria, despite the introduction of DOTS decades ago, the average rate of successful treatment is 75.3% (Kwaghe et al. 2020). Previous studies reported varied TB treatment success rates of 83.7%, 86%, 85.7%, and 67.4%, in Kaduna, Anambra, Lagos Plateau, and Ebonyi (Adejumo et al. 2016; Akanbi et al. 2019; Alobu et al. 2014; Babatunde et al. 2016; Daniel and Alausa 2006). Similar studies documented treatment success rates of 56.5%, 81.9%, 80%, 76.6% and 83.5% in Sokoto, Bauchi, Oyo, and Ogun states, respectively (Gidado and Ejembi 2009; Kwaghe et al. 2020; Oladimeji et al. 2021; Oshi et al. 2020; Sariem et al. 2020; Umeokonkwo et al. 2020). Some of these results are below the WHO-recommended threshold of 85–90% success rates (Chakaya et al. 2021; Kwaghe et al. 2020). Because a substantial number of pulmonary TB cases were not registered and enroled into the DOTS by the Nigerian National TB control program for treatment. This creates a treatment gap at the referral centres, which sequentially affects the control and prevention of TB, the treatment outcome and its determinants (Kwaghe et al. 2020).

A higher rate of successful treatment outcomes has reduced the TB burden and mortality rate in the population. An unsuccessful treatment outcome result has an immediate effect on the socioeconomic well-being of the population. Clinically, it will fast-track the spread of multi-drug resistance pulmonary tuberculosis in the community (Danlami et al. 2021; Oladimeji et al. 2021). Therefore, monitoring and evaluating the successful and unsuccessful treatment outcomes of pulmonary tuberculosis in the government’s referral centres are integral parts of an effective TB control program in Nigeria. This study investigated tuberculosis treatment outcomes and the factors associated with unsuccessful outcomes never assessed in the 21 referral hospitals in Kebbi State, Nigeria.

## Methods

Kebbi with a population of approximately 4.4 million is the tenth largest and the 22nd most populous state in Nigeria. The state has 21 Local Government Area Councils (LGA). The prospective pulmonary TB data from DOTS facilities in the LGAs are collected by the LGA TB focal persons and submitted to the State TB and Leprosy Control Managers, who report to the National TB and Leprosy Control Program (NTBLCP) (Oladimeji et al. 2021).

### Study design

The study reviewed treatment outcomes of pulmonary TB patients managed in the DOTS facilities of the 21 general hospitals of Kebbi State between January 2018 and December 2021. The socio-demographic data retrieved from the patient records were the year of infection, age and sex. The clinical data retrieved from the patient data were the disease classification, DOTS facility, HIV status, diagnosis, and case management type.

### Inclusion criteria

The criteria adopted in the study included pulmonary TB patients between 5–70 years of age who started TB treatment in any of the 21 general hospitals with DOTS facilities between January 2018 and December 2021. All combined and confirmed pulmonary TB and extrapulmonary TB infections were included in the study. Pulmonary TB patients with confirmed multi-drug resistance tuberculosis with no definitive treatment strategy were excluded from the study.

### Key terms/definition

*Cured*: A cured patient is a confirmed smear-positive patient at the start of treatment who was smear negative six months after the treatment commences or at the end of treatment.

*Treatment completed* This is a pulmonary TB patient who begins treatment but without a documented record of smear or culture-negative results of treatment.

*Treatment failure* A pulmonary TB treatment failure is a patient who commenced treatment but with a smear or culture-positive result during or five months after the commencement of the treatment.

*Died* A pulmonary TB patient who commenced treatment and dies before completing the treatment.

*Defaulted* A pulmonary TB patient has defaulted if he did not commence treatment and or commenced the treatment but interrupted for two successive months or more.

*Loss to follow-up* A pulmonary TB patient with unknown records of treatment outcomes due to transfer to other DOTS facilities.

*Treatment success* A pulmonary TB patient with successful treatment is a patient with a smear-positive result at the beginning of treatment but cured and completes the treatment with a confirmed smear-negative result.

### Data collection and analysis

The research assistant collated the data between January 2018 and December 2021. Each patient's data were entered and coded into Microsoft Excel 1 version 2013. The coded data were analysed using Statistical Package for the Social Sciences (SPSS®, version 21; SPSS Inc., Chicago, IL, USA). Descriptive analysis was adopted to identify associations among variables. Logistic regression was used to analyze the relationship between the dependent and independent variables. The association between variables was presented as the odds ratio and adjusted odd ratio with nominal 95% confidence intervals (CI) at a *p*-value of 0.05.

## Results

The study reviewed 6114 documented records of PTB patients. 1161 (18.9%) commenced treatment. 963 (82.9%) were males and 198 (17.1%) were females. 985 (18.2%) of TB patients reviewed had documented treatment outcomes. 64 (5.8%) of the patient with documented treatment outcomes were HIV seropositive and 932 (95.1%) had a pulmonary infection (Table 1). Of the TB patients reviewed, 903 (91.7%) treatments were successful, and 82 (8.3%) failed. Among the successful treatment outcome, 411 (41.7%) were cured, and 492 (49.9%) completed the treatment. Of the patients with unsuccessful treatment outcomes, 15 (1.5%) lost-to-follow-up, 43 (4.4%) defaulted and 24 (2.4%) died. The highest rate of successful treatment was recorded in females (97.6%), patients with new cases (92.3%) and with pulmonary infections (91.2%) (Table 2).

**Table 1:** Socio-demographic and clinical characteristics of TB patients attending the DOTS facilities in 21 referral hospitals in Kebbi State

Variables	Frequency	Percentage (%)
*Age*		
Less than 20	93	9.2
21–40	484	47.6
41–60	197	19.3
Above 60	242	23.9
*Year*		
2018	59	5
2019	89	7.5
2020	486	41.2
2021	546	46.3
*Sex*		
Male	963	82.9
Female	198	17.1
*Type of infection*		
Pulmonary	932	95.1
Extra pulmonary	48	4.9
*Diagnosis*		
AFB	693	59.3
GeneXpert	474	40.7
*DOTA support*		
Yes	763	69.7
No	332	30.3
*Treatment success*		
Successful	903	91.7
Unsuccessful	82	8.3
*HIV/TB status*		
Positive	64	5.8
Negative	1036	94.2
*Types of patients*		
New	1088	92.6
Old TB cases	87	7.4

**Table 2:** Treatment outcomes of TB patients attending the DOTS facilities in 21 referral hospitals in Kebbi State (*N* = 1161)

Variables	Frequency	Percentage
Cured cases	411	41.7
Treatment completed	492	49.9
Death	24	2.4
Defaulted	43	4.4
Lost-to-follow-up	15	1.5
Treatment failure	82	8.3
Treatment success	903	91.7

The risk of treatment failure among the elderly was 0.90 (95% CI 0.48–1.68) higher compared to those aged 15–40 years. Positive HIV status had a 1.91, 95% CI (0.59–6.23) times greater risk of unsuccessful treatment outcome compared to those with negative HIV status. Patients with old TB cases had a 2.43, 95% CI (1.28–4.63) times greater than new cases (Table 3). In multivariate logistic analysis, the odds of treatment failure were two-fold higher among patients with extrapulmonary infections (AOR = 2.40, 95% CI 1.12–5.39), PTB elderly patients above 40 years of age (AOR = 2.00, 95% CI 1.37–2.92), and three-fold higher with old or other cases (AOR = 3.03, 95% CI 1.47–7.19) as compared to their groups. Overall, the likelihood of patients having successful treatment outcomes in a DOTS facility is (AOR = 0.52, 95% CI 0.42–0.77) (Table 3).

**Table 3:** Logistic regression analyses of factors associated with treatment outcomes in TB patients attending the DOTS facilities in 21 referral hospitals in Kebbi State

Variables	Treatment outcome		95 Confidence interval		*P* value
	Yes (903)	No (82)	COR (95% CI)	AOR (95% CI)	
*Age*					
Less than 40 years	385	25	Ref	Ref	
Greater than 40 years	518	57	1.69 (1.04–2.76)	2.00 (1.37–2.92)	0.744
*Year*					
2018–2019	131	13	Ref	Ref	
2020–2021	772	69	0.90 (0.48–1.68)	0.06 (0.32–1.62)	0.043*
*Sex*					
Male	799	79	Ref	Ref	
Female	104	3	0.29 (0.09–0.94)	0.22 (0.11–0.74)	0.025*
*Infection*					
Pulmonary	882	78	Ref	Ref	
Extra pulmonary	21	4	2.15 (0.72–6.43)	2.40 (1.12–5.39)	0.149
*Diagnosis*					
AFB	546	56	Ref	Ref	
GeneXpert	357	26	0.71 (0.44–1.15)	0.88 (0.76–1.25)	0.193
*DOTS support*					
Yes	573	66	Ref	Ref	
No	330	16	0.42 (0.24–0.74)	0.52 (0.42–0.77)	0.002*
*HIV/TB status*					
Positive	61	3	Ref	Ref	
Negative	842	79	1.91 (0.59–6.23)	2.24 (0.52–9.68)	0.20
*Types of patients*					
New	838	69	Ref	Ref	
Old TB cases	65	13	2.43 (1.28–4.63)	3.03 (1.47–7.19)	0.009*

*Statistically significant (*p* < 0.05) Ref = Reference

## Discussion

Herein a retrospective study, the overall rate of successful treatment among TB patients attending public hospitals in Kebbi State is 91.7%. This rate is slightly higher than the success rate of 85% recommended by the world health organization (Harding 2020). This result is similarly higher than the targeted treatment success rate of 86% among all new TB cases reported around the world and in the Anambra and Lagos states in Nigeria (Kwaghe et al. 2020; Oladimeji et al. 2021; Umeokonkwo et al. 2020). The success rate reported in this retrospective study, however, is in contrast to the mean annual successful treatment rate of 75.3% reported in Nigeria and several states in the country (Kwaghe et al. 2020). The variation in the successful treatment rate could be attributed to factors associated with the research design and the number of respondents in the study population, the quality of the DOTS strategy and facilities (Jacobson et al. 2015), and the patient TB-HIV co-infected status (Gebrezgabiher et al. 2016). The study follows up on patients who contracted the disease pre-COVID-19 (2018–2019) and post-COVID-19 (2020–2021). Despite this, the anticipated effects of COVID-19-related disruptions in access to TB care were not considered, because the most significant impact of the pandemic on access to DOT facilities, was lockdown. The current study area is among the few in the country with few cases of COVID-19 (480 cases, 16 deaths) (NCDC 2022), partial lockdown and unrestricted movement of people including TB patients who need access to diagnostic facilities and treatment centres.

This study revealed that the majority of the pulmonary TB cases reviewed were males because a high percentage of males were predisposed to pulmonary tuberculosis due to their social habits, practices, and occupations (Danlami et al. 2021). The women are prone to pulmonary tuberculosis due to their caregiver roles and social status, thus a reduced frequency of women than men. In addition, women are more likely to seek medical care early from certified healthcare facilities. Therefore, their role in the community involves protecting their children and families from infection (Danlami et al. 2021). Seeking medical care early helps prevent diseases and improve quality of life. Correspondingly, one-fourth (80.1%) of the total patients reviewed were not documented for treatment. This might be connected with stigmatization towards TB patients and its gender specific. Male TB patients had a penchant for keeping information or providing wrong information about their TB status to avoid visits or follow-up calls from healthcare providers. They constituted the majority of cases for whom the treatment outcome is unknown to the reporting unit. Whereas in female patients, despite stigmatization in the form of abandonment and emotional abuse, information about their TB status is open to families and friends. They welcome, visit and embrace health caregivers and the evidence is clear from the success recorded in the treatment outcome in this study. The reduced number of TB patients not utilizing the DOTS service contributed to highly successful treatment outcomes and low treatment failure in the study. The failure of the patient to present themselves for treatment might be associated with the proximity of referral hospitals for the patients (Babatunde et al. 2016; Jacobson et al. 2015), lack of awareness, and poor financial status (Chakaya et al. 2022; Kwaghe et al. 2020; Umeokonkwo et al. 2020). The study also observed a lower rate of unsuccessful treatment outcomes 8.3% in the study area, which might be a result of cooperation between the DOTS supervisors and the documented TB patients in good adherence to TB treatment (Alobu et al. 2014; Babatunde et al. 2016). The high percentage of treatment failure reported due to loss of follow-up and death is 4.4% and 2.4%, respectively. In previous studies, the rate of unsuccessful treatment outcomes due to loss of follow-up increases the risk of death due to the incapacitation of TB patients occasioned by the multi-drug resistant strains of *M. tuberculosis* (Chakaya et al. 2021; Gidado and Ejembi 2009; Oshi et al. 2020).

In the logistic regression analysis, older age, HIV status, patients with old cases and extrapulmonary infections were the predisposing factors to unsuccessful treatment outcomes among TB patients. Consistent with previous findings, the risk of having unsuccessful treatment outcome were more pronounced in elderly patients due to changes in body composition in old age, unable to adhere to treatment due to complex regimens and multiple prescribing, and low immunity due to ongoing treatment (Adejumo et al. 2016; Chakaya et al. 2021; Hall et al. 2017). Similarly, patients with immune suppression due to HIV seropositive have reported poor treatment outcomes due to the depletion and dysfunction of CD4 cells by HIV that weakens the body system from fighting infection (Akanbi et al. 2019; Babatunde et al. 2016; Daniel and Alausa 2006; Eshetie et al. 2018). The old TB cases in this study are patients who have had TB treatment and whose treatment failed at the end of their most recent course of treatment. The unsuccessful treatment outcomes observed among old TB cases might be either to the emergence of drug resistance during the treatment course or underlying disease conditions (Danlami et al. 2021; Loveday et al. 2012; Sariem et al. 2020). Additionally, the present study showed that patients with combined pulmonary TB and extrapulmonary infections had lower treatment success rates compared to pulmonary TB patients (Atif et al. 2018; Eshetie et al. 2018; Hall et al. 2017). This might be connected with the effects of combined pulmonary TB and extrapulmonary infections as well as the HIV status of the patient in this group, as a significant number of the TB patients with extrapulmonary infections had TB-HIV co-infection. Patients in this group are more likely to have lower treatment success rates or fail treatment than patients with negative HIV status (Loveday et al. 2012; Oladimeji et al. 2021).

## Conclusions

The study reported a treatment success rate of 91.7% among TB patients attending public hospitals in Kebbi State. The outcome was higher than the projected success rate of 85% set by the WHO. The result from this study is attributed to good adherence to TB treatment, which highlighted the performance of institutional DOTS in the study area. Nonetheless, one-fourth of the total patients reviewed were not documented for treatment, this contributed to a lower number of patients with unsuccessful treatment outcomes. The high percentage of TB patients not enroled on DOTS facilities could be a reservoir of the high TB burden in the area. Therefore, the study recommends the need for designing effective tracing methods to identify and enrol patients for effective and successful TB treatment outcomes in the study area and the country.

## Data Availability

The datasets are available from the corresponding author upon request.

## Abbreviations

AOR: Adjusted Odd Ration
COR: Corrected Odd Ratio
CD4: Cluster of Differentiation 4
CI: Confidence Intervals
DOTS: Directly Observed Therapy
DOTS: Directly Observed Therapy Short-course
HIV: Human Immunodeficiency Virus
LGA: Local Government Authority
NTBLCP: National TB and Leprosy Control Program
PTB: Pulmonary Tuberculosis
P.M.B.: Private Mail Box
TB: Tuberculosis
TB-HIV: Tuberculosis- Human Immunodeficiency Virus
WHO: World Health Organization

## References

[CR1] Adejumo OA, Daniel OJ, Adebayo BI, Adejumo EN, Jaiyesimi EO, Akang G, Awe A (2016). Treatment outcomes of childhood TB in Lagos, Nigeria. J Trop Pediatr.

[CR2] Akanbi K, Ajayi I, Fayemiwo S, Gidado S, Oladimeji A, Nsubuga P (2019). Predictors of tuberculosis treatment success among TB-HIVco-infected patients attending major tuberculosis treatment sites in Abeokuta, Ogun State, Nigeria. Pan Afr Med J.

[CR3] Alobu I, Oshi SN, Oshi DC, Ukwaja KN (2014). Risk factors of treatment default and death among tuberculosis patients in a resource-limited setting. Asian Pac J Trop Med.

[CR4] Atif M, Anwar Z, Fatima RK, Malik I, Asghar S, Scahill S (2018). Analysis of tuberculosis treatment outcomes among pulmonary tuberculosis patients in Bahawalpur, Pakistan. BMC Res Notes.

[CR5] Babatunde OI, Christiandolus EO, Bismarck EC, Emmanuel OI, Chike AC, Gabriel EI (2016). Five years retrospective cohort analysis of treatment outcomes of TB-HIV patients at a PEPFAR/DOTS Centre in South Eastern Nigeria. Afr Health Sci.

[CR6] Chakaya J, Khan M, Ntoumi F, Aklillu E, Fatima R, Mwaba P, Kapata N, Mfinanga S, Hasnain SE, Katoto PD, Bulabula AN (2021). Global Tuberculosis Report 2020–Reflections on the Global TB burden, treatment and prevention efforts. Int J Infect Dis.

[CR7] Chakaya J, Petersen E, Nantanda R, Mungai BN, Migliori GB, Amanullah F, Lungu P, Ntoumi F, Kumarasamy N, Maeurer M, Zumla A (2022). The WHO Global Tuberculosis 2021 Report–not-so-good news and turning the tide back to End TB. Int J Infect Dis.

[CR8] Daniel OJ, Alausa OK (2006). Treatment outcome of TB/HIV positive and TB/HIV negative patients on directly observed treatment, short course (DOTS) in Sagamu, Nigeria. Niger J Med.

[CR9] Danlami MB, Aliyu B, Samuel G (2021). Incidence of rifampicin-resistance presumptive *M. Tuberculosis* cases among outpatients in Kebbi State, Nigeria. Afr J Infect Dis.

[CR10] Eshetie S, Gizachew M, Alebel A, van Soolingen D (2018). Tuberculosis treatment outcomes in Ethiopia from 2003 to 2016, and impact of HIV co-infection and prior drug exposure: a systematic review and meta-analysis. PLoS ONE.

[CR11] Gebrezgabiher G, Romha G, Ejeta E, Asebe G, Zemene E, Ameni G (2016). Treatment outcome of tuberculosis patients under directly observed treatment short course and factors affecting outcome in Southern Ethiopia: a five-year retrospective study. PLoS ONE.

[CR12] Gidado M, Ejembi CL (2009). Tuberculosis case management and treatment outcome: assessment of the effectiveness of Public-Private Mix of tuberculosis programme in Kaduna State, Nigeria. Ann Afr Med.

[CR13] Hall EW, Morris SB, Moore BK, Erasmus L, Odendaal R, Menzies H, van der Walt M, Smith SE (2017). Treatment outcomes of children with HIV infection and drug-resistant TB in three provinces in South Africa, 2005–2008. Pediatr Infect Dis J.

[CR14] Harding E (2020). WHO global progress report on tuberculosis elimination. Lancet Respir Med.

[CR15] Jacobson KB, Moll AP, Friedland GH, Shenoi SV (2015). Successful tuberculosis treatment outcomes among HIV/TB coinfected patients down-referred from a district hospital to primary health clinics in rural South Africa. PLoS ONE.

[CR16] Kwaghe AV, Umeokonkwo CD, Aworh MK (2020). Evaluation of the national tuberculosis surveillance and response systems, 2018 to 2019: National Tuberculosis, Leprosy and Buruli Ulcer Control Programme, Abuja, Nigeria. Pan Afr Med J.

[CR17] Loveday M, Wallengren K, Voce A, Margot B, Reddy T, Master I, Brust J, Chaiyachati K, Padayatchi N (2012). Comparing early treatment outcomes of MDR-TB in decentralised and centralised settings in KwaZulu-Natal, South Africa. Int J Tuberc Lung Dis.

[CR18] NCDC (2022) COVID-19 Nigeria. Nigeria Centre for Disease Control. https://covid19.ncdc.gov.ng/

[CR19] Oladimeji O, Adepoju V, Anyiam FE, San JE, Odugbemi BA, Hyera FL, Sibiya MN, Yaya S, Zoakah AI, Lawson L (2021). Treatment outcomes of drug-susceptible Tuberculosis in private health facilities in Lagos, South-West Nigeria. PLoS ONE.

[CR20] Oshi D, Chukwu J, Nwafor C, Chukwu NE, Meka AO, Anyim M, Ukwaja KN, Alobu I, Ekeke N, Oshi SN (2020). Support and unmet needs of patients undergoing multidrug-resistant tuberculosis (MDR-TB) treatment in southern Nigeria. Int J Health Plann Manag.

[CR21] Sariem CN, Odumosu P, Dapar MP, Musa J, Ibrahim L, Aguiyi J (2020). Tuberculosis treatment outcomes: a fifteen-year retrospective study in Jos-North and Mangu, Plateau State, North-Central Nigeria. BMC Public Health.

[CR22] Umeokonkwo CD, Okedo-Alex IN, Azuogu BN, Utulu R, Adeke AS, Disu YO (2020). Trend and determinants of tuberculosis treatment outcome in a tertiary hospital in Southeast Nigeria. J Infect Public Health.

